# Antagonizing Bcl-2′s BH4 domain in cancer

**DOI:** 10.18632/aging.100828

**Published:** 2015-10-22

**Authors:** Tamara Vervloessem, Rita La Rovere, Geert Bultynck

**Affiliations:** KU Leuven, Laboratory of Molecular and Cellular Signaling, Department of Cellular and Molecular Medicine, Campus Gasthuisberg O/N-I bus 802, Leuven, Belgium

**Keywords:** Bcl-2 inhibitors, BH4 domain, apoptosis, anticancer, peptides, small molecules

**Figure 1:**
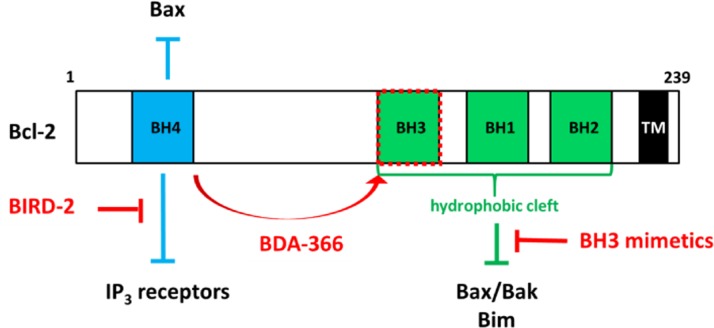
Two functional domains, the BH4 domain and the hydrophobic cleft, are important for Bcl-2's anti-apoptotic function. The transmembrane (TM) domain anchors Bcl-2 in ER and mitochondrial membranes. The BH4 domain suppresses apoptosis by binding and inhibiting Bax (in mitochondria) and IP3 receptors (in ER). The hydrophobic cleft interacts with several pro-apoptotic Bcl-2 family members, including Bax/Bak and BH3-only proteins like Bim. BH3 mimetics, like ABT-737, ABT-263 and ABT-199, target the hydrophobic cleft of Bcl-2 and release Bim, leading to Bim-mediated activation of Bax/Bak and inducing apoptosis. Furthermore, BIRD-2 (Bcl-2/IP_3_ receptor Disruptor-2) and BDA -366 have been developed to antagonize Bcl-2 via its BH4 domain leading to apoptosis although via different mechanisms. BIRD-2 provokes pro-apoptotic Ca^2+^ signaling, while BDA-366 causes a conformational change in Bcl-2, resulting in the exposure of its BH3 domain, which will activate Bax

Many cancer cells, including B-cell and lung cancers, display elevated expression of anti-apoptotic Bcl-2 proteins as a survival strategy to cope with oncogenic stress [1]. In cancer cells, Bcl-2 is loaded with proapoptotic BH3-only proteins, thereby not only preventing Bax/Bak activation but also rendering them “primed to death” at the mitochondria. The last decade, different compounds have been developed to antagonize this anti-apoptotic function of Bcl-2 at the mitochondria [1]. The most promising molecules are the BH3 mimetics (like ABT-737 and ABT-263), which release Bim from the hydrophobic cleft of Bcl-2 (or Bcl-XL) formed by the BH3-BH1-BH2 domains resulting in Bax/Bak-mediated apoptosis in cancer but not in healthy cells (Fig. 1). The last generation BH3 mimetics (ABT-199) spares platelets by avoiding Bcl-XL inhibition [1].

Nowadays, the BH4 domain of Bcl-2 has emerged as an important anti-apoptotic mechanism by preventing Bax activation [2] and by inhibiting IP_3_ receptors, a major class of intracellular Ca^2+^-release channels involved in cell death and survival [3]. Importantly, cancer cells appear to exploit this function of Bcl-2 to prevent proapoptotic Ca^2+^ release from the endoplasmic reticulum and mitochondrial Ca^2+^ overload. Recently, a stabilized cell-permeable IP_3_R-derived peptide, BIRD-2 (Bcl-2/IP_3_ receptor Disruptor-2) was developed by Distelhorst and co-workers (Fig. 1). This peptide provoked, by antagonizing the BH4 domain of Bcl-2, pro-apoptotic Ca^2+^ signaling in a variety of lymphoid malignancies: primary chronic lymphocytic leukemia (CLL) and diffuse large B-cell lymphoma (DLBCL) cells (reviewed in [3]) and in multiple myeloma and follicular lymphoma cells [4]. BIRD-2-induced cell death, which involves Bax and caspase 3 activation, also resulted in a marked decrease in tumor growth in in vivo xenograft mouse [4]. Importantly, BIRD-2 did not cause a general cytotoxicity as peripheral mononuclear blood cells, certain DLBCL cells and non-malignant cell lines were very resistant to BIRD-2. Susceptibility of cancer cells to BIRD-2 was linked in DLBCL cell lines to the expression of the type 2 IP_3_ receptor, the isoform with the highest sensitivity towards its ligand IP_3_ (reviewed in [3]). Moreover, cancer cells that were more sensitive to BIRD-2 appeared more resistant to BH3 mimetics and vice versa [4]. This is important since cancers poorly responding to conventional chemotherapy are also poor responders to BH3 mimetics, as both responses depend on the “mitochondrial apoptotic priming” status [5]. Interestingly, Distelhorst and co-workers recently showed that prolonged exposure of myeloma cells to BIRD-2 elevated Bim-protein levels via a Ca^2+^-dependent mechanism, thereby increasing their sensitivity to BH3-mimetics and inducing synergistic effects with these drugs [4]. Correlating with this, in BIRD-2-sensitive DLBCL cells, BIRD-2 could boost cell death provoked by HA14-1, a Bcl-2 inhibitor that also impacts Ca^2+^ signaling by inhibiting the sarco/endoplasmic reticulum Ca^2+^ ATPase [6].

Yet, the therapeutic applicability of native peptides in humans may be limited due to issues with (oral) bioavailability and stability. Hence, Deng and co-workers identified, by screening chemical compounds, BDA-366 that binds with very high affinity (Kd of ∼3.3 nM) and selectivity to the BH4 domain of Bcl-2, but not to Bcl-XL, Mcl-1 or Bfl-1 [7]. BDA-366 induced a conformational change in Bcl-2, resulting in the exposure of its BH3 domain that binds and activates Bax indirectly, leading to apoptosis (Fig. 1). Hence, BDA-366 resulted in cell death in different lung cancer cell lines with high levels of endogenous Bcl-2 but not in those with low or zero Bcl-2 levels. BDA-366, applied intraperitoneally, also prevented the growth of lung cancer cell lines and patients cells in in vivo xenograft mouse models without causing toxicity in healthy tissues. Moreover, current anti-lung cancer therapies inhibit mTOR, which adversely causes an upregulation of endogenous Bcl-2 levels. Importantly, BDA-366 displayed synergistic action with RAD001, an mTOR inhibitor currently used in the clinic for treating lung cancer.

BDA-366 also disrupted Bcl-2 / IP_3_ receptor complexes and increased Fura-2 fluorescence, suggesting an effect on Ca^2+^ signaling. However, the impact of BDA-366 on Ca^2+^ dynamics and on interaction of Bcl-2 with IP_3_ receptors as well as on the importance of Ca^2+^ signaling for BDA-366-induced cell death in normal and malignant cells require further study. Furthermore, it remains unknown whether other BH4-domain-targeting molecules can impact the conformation of Bcl-2. Moreover, by switching Bcl-2 into a pro-apoptotic protein with an exposed BH3 domain, BDA-366 also results in the release of “activator” BH3-only proteins, like Bim, but their importance for BDA-366-induced apoptosis ought to be further characterized. Thus, facilitating the release of Bim using BH3 mimetics or boosting the expression of Bim using BIRD-2 might present interesting avenues to enhance BDA-366-induced cell death. Further challenges towards its clinical applicability will also include (i) characterizing its effects in other Bcl-2-dependent cancers, (ii) examining its ability to boost other anti-cancer strategies currently used to treat these cancers (like chemotherapy, antibody therapy and phototherapy) and (iii) documenting its therapeutic window, thereby avoiding the potential adverse effects of such compounds on native, healthy tissues that express high endogenous levels of Bcl-2, including the hematopoietic system, endocrine glands like the thyroid gland, respiratory system like nasopharynx, reproductive systems, kidney and bladder (http://www.proteinatlas.org/ENSG00000171791-CL2/tissue). In conclusion, these recent studies [4, 7] reveal that antagonizing Bcl-2 via its BH4 domain holds potential for future cell-death therapies in different cancers, including those resistant to conventional chemotherapy/BH3 mimetics.
